# Supplementary material for: A Pilot Survey: Oral Function as One of the Risk Factors for Physical Frailty
Source: Int J Environ Res Public Health. 2022 May 18;19(10):6136. doi: 10.3390/ijerph19106136 (PMC9141329; doi:10.3390/ijerph19106136)
Supplement: Supplementary file 1 [file ijerph-19-06136-s001.zip › ijerph-1701378-supplementary.pdf]

Supplementary Table S1. Questions of Eleven Check.

**Eating habits**
Do you try to eat healthier than persons of the same gender and age?
Do you eat both a vegetable dish and a main dish (meat or fish) at least twice daily?
**Oral functions**
Can you chew through even hard foods, such as "dried squid" and "pickled radish" normally?
Do you choke on tea or soup?
**Motor functions**
Do you exercise to the point of sweating for at least 30 minutes a day at least twice a week and more than a year?
Do you walk or engage in similar physical activity in your daily life for at least one hour a day?
Do you think you can walk faster than persons of the same gender and age?
**Social and mental functions**
Do you go out less often than you did last year?
Do you eat with others at least once a day?
Do you consider yourself to be vibrant?
Are you worried about forgetfulness more than anything else?
